# Risk factors of hikikomori among office workers during the COVID-19 pandemic: A prospective online survey

**DOI:** 10.1007/s12144-022-03446-8

**Published:** 2022-07-29

**Authors:** Hiroaki Kubo, Ryoko Katsuki, Kazumasa Horie, Itsuki Yamakawa, Masaru Tateno, Naotaka Shinfuku, Norman Sartorius, Shinji Sakamoto, Takahiro A. Kato

**Affiliations:** 1 Department of Neuropsychiatry, Graduate School of Medical Sciences, Kyushu University, 3-1-1 Maidashi Higashi-Ku, Fukuoka, 812-8582 Japan; 2 Department of Health and Human Services, Faculty of Medical and Welfare, Tohoku Bunka Gakuen University, Miyagi, Japan; 3 Department of Psychology, College of Humanities and Sciences, Nihon University, Tokyo, Japan; 4 Tokiwa Child Development Center, Tokiwa Hospital, Sapporo, Japan; 5 Department of Neuropsychiatry, School of Medicine, Sapporo Medical University, Sapporo, Japan; 6 School of Human Sciences, Seinan Gakuin University, Fukuoka, Japan; 7 Association for the Improvement of Mental Health Programmes, Geneva, Switzerland

**Keywords:** Hikikomori (pathological social withdrawal), Modern-type depression (MTD), Self-restraint behavior, Office workers, COVID-19 pandemic

## Abstract

The global pandemic of COVID-19 has forced people to restrict their outings. In Japan, self-restraint behavior (SRB) has been requested by the government, and some of those decreasing their outings may shift to pathological social withdrawal; hikikomori. The purpose of this study was to examine the risk factors of hikikomori conducting an online prospective survey. An online survey was conducted in June 2020 and December 2020; (1) SRB-related indicators (degree of SRB, motivation for SRB, stigma and self-stigma toward COVID-19, anxiety and depressive feelings toward COVID-19) and (2) general mental health (hikikomori tendency, depressive symptoms, modern type depression (MTD) tendency, internet addiction) were collected. A cross-lagged effects model was performed to examine the association between these variables. Lack of emotional support and lack of socialization in June 2020 increased isolation in December 2020. Besides, MTD and hikikomori interacted with each other. Interestingly, although hikikomori tendency increased depressive tendencies, SRB itself did not have a significant path on any mental health-related variables. Poor interpersonal relationships, rather than SRB per se, are suggested to be a risk factor for increased isolation among office workers in the COVID-19 pandemic. Appropriate early interventions such as interpersonal or emotional support may prevent the transition to pathological hikikomori. The association between MTD and hikikomori seems to reveal the interesting possibility that MTD is a gateway to increased risk of hikikomori, and that hikikomori is a gateway to MTD as well. Future research is required to elucidate the relationship between hikikomori and MTD.

## Introduction

Hikikomori is a condition of staying at home almost every day for more than six months with avoidance of going out or participating in society (Saito, 2010). Originally, hikikomori had been thought to be a Japanese culturally-based syndrome associated with avoidance of stressful situations, causing significant distress and disability (Kato et al., 2011a, b, 2018; Teo et al., 2015a). People with hikikomori spend most of the day at home almost every day; on the other hand, not a few people with hikikomori can go out where interpersonal interaction is not necessary. Thus, those who go out for limited periods of less than an hour (e.g., going out for shopping late at night) are also included in hikikomori (Kato et al., 2020a; Saito, 2010; Teo et al., 2015a). The number of people with hikikomori is estimated to be more than 1.1 million in Japan (Kato et al., 2019a, b). Now, hikikomori cases are also reported in many other countries around the world, and hikikomori is becoming a global issue (Kato et al., 2012, a, b; Wu et al., 2019). Hikikomori is believed to be associated with amae (Doi, 1971). Amae is dependent behavior based on the expectation that caregivers will tolerate such dependency. In the case of hikikomori, the situation is facilitated by parents’ acceptance of staying at home for a long time (Kato et al., 2012). Originally, amae has been thought to be unique to Japanese culture, however it has been suggested to be universal in other cultures as well (Kato et al., 2012). Hikikomori results in serious problems in education, and at work and is comorbid with mental health problems (Kato et al., 2019a, b). Until now, no effective treatment methods have been developed (Kato et al., 2018, a, b). Several studies on hikikomori and psychiatric diagnoses show that a wide range of comorbidity are reported such as anxiety disorders, depression, schizophrenia, personality disorders, and developmental disorders (Kato et al., 2012; Kondo et al, 2013; Koyama et al., 2010; Teo et al., 2015b). Several epidemiological studies have been conducted to elucidate hikikomori (Imai et al., 2020; Malagón-Amor et al., 2020; Nonaka & Sakai, 2021; Yong & Nomura, 2019). Risk factors for hikikomori include introversion combined with stressful events (Chong & Chan, 2012), interpersonal problems (Yong & Nomura, 2019), and low self-esteem (Chan & Lo, 2014). Lack of motivation to conform to the social mainstream and dropout from education are also assumed to result in hikikomori (Norasakkunkit & Uchida, 2014; Yong & Nomura, 2019). However, given the recent rapid changes in social conditions, i.e., COVID-19 pandemic, forcing us to be in hikikomori-like condition, new research would be required to confirm risk factors of hikikomori related to such global changes (Kato et al., 2020b).

The global pandemic of COVID-19 continues to be major health problem in most parts of the world. Many governments are taking measures to combat the infection, by measures such as confinement to homes, restrictions of travel, and closing workplaces to ensure physical distance and limit the flow of people (Gostin & Wiley, 2020). There is a concern that these restrictions may negatively impact mental health. The impacts of confinement to homes on mental health such as depression and anxiety, health and financial insecurity, alcohol use, spousal violence and loneliness are reported by many sources (Hamadani et al., 2020; Neill et al., 2020; Tull et al., 2020; Ueda et al., 2020). Forced isolation and prolonged restrictions on going out may lead to more serious mental health problems such as pathological social withdrawal; hikikomori (Kato et al., 2020b). Hikikomori is considered to be a coping behavior avoiding anxiety situations (Kato et al., 2012; Tateno et al., 2012; Li & Wong, 2015; Malagón-Amor et al. 2015; Kato et al., 2016a, b; 2020c). In addition, deficiency in functional and instrumental coping skills such as help-seeking is suggested to increase hikikomori-like behaviors, leading to more severe hikikomori (Kondo et al., 2013; Nonaka & Sakai, 2021). In the COVID-19 pandemic, poor psychological flexibility is suggested to be associated with nonfunctional coping such as alcohol consumption or even agoraphobia (Akbari et al., 2021; Kato et al., 2020b). Increasing flexibility and developing appropriate coping skills may help prevent or improve hikikomori (Akbari et al., 2021; Nonaka & Sakai, 2021).

Unlike other countries, the Japanese government has not implemented enforceable measures such as lockdowns or home confinement orders. Instead, Japanese people are required to refrain from going out without legal binding power (i.e., they are expected to practice self-restraint behavior; SRB). In other words, people are not sanctioned or arrested legally, but are expected to conduct themselves in accordance with government-announcing behavioral restrictions. Due to the unclear end to the massive COVID-19 pandemic, the Japanese government has been repeatedly declaring the state of emergency, and as a result, people continue to be asked to SRB for a long time. Although SRB may cause hikikomori, to the best of our knowledge, no study has been conducted to examine the relationship between SRB and hikikomori.

On the other hand, epidemiological studies of hikikomori reported that mental health problems such as depression, modern-type depression (MTD) (Teo et al., 2020), and Internet addiction (Tateno et al., 2019) are also associated with hikikomori. MTD is a condition characterized by situation-dependent depressive symptoms and avoidant tendencies, which has recently been observed mainly in young adults in Japan (Kato & Kanba, 2017; Kato et al., 2011a, b, 2016a, b). Lack of motivation to fulfill roles expected by society in MTD seems to be similar to hikikomori, and low self-esteem has been reported in MTD as in hikikomori (Kato et al., 2019a, b). A case–control study comparing depressive patients with and without hikikomori reported that MTD tendencies were found to be higher in patients with hikikomori (Teo et al., 2020). Furthermore, a therapeutic intervention for hikikomori has been shown to decrease social anxiety and increase self-esteem (Wong et al., 2019). These findings suggest that in the midst of a large-scale COVID-19 pandemic, it will be necessary to explore the risk of hikikomori in relation to SRB but also mental health problems such as depression, MTD, and Internet dependence, as well as social anxiety, motivation, and self-esteem. Detailed examination of these associations is expected not only to prevent hikikomori in the pandemic, but also to systematically identify risk factors for hikikomori. Therefore, the present study aimed to simultaneously examine the relationship between SRB and mental health problems such as depression, MTD, and Internet addiction, and hikikomori by a prospective online survey.

## Methods

This study was conducted in accordance with the Declaration of Helsinki and was approved by the ethics committees of Kyushu University and Nihon University.

### Participants

The participants in the present study were part of the 1053 participants in our previous online survey (Katsuki et al., 2021). We asked those 1053 participants to join in the second survey conducted six months later; 247 did not respond to our request and 806 agreed. Among them, 800 participants with no missing values were included in the present study. They resided in all prefectures in Japan. Participants were recruited through an online survey company (Cross Marketing Inc., Tokyo, Japan). Eligibility criteria were age between 30 and 59 years old, and ability to understand Japanese. In addition, participants were recruited so that the gender ratio was equal in each age group (30 s, 40 s, and 50 s). In recruitment, participants were informed of the study: the survey was anonymous and completely voluntary. They agreed to participate in the study with informed consent.

### Data collection

The online survey was conducted twice with a six-month interval between the two surveys. The first survey was conducted in June 2020 to obtain baseline data on SRB-related indicators and mental health associated with hikikomori. The second survey was conducted in December 2020 to obtain data on hikikomori (actual going out and hikikomori tendency) and mental health. Each questionnaire is described below.

### Measurements

#### (1) Questionnaires implemented in June 2020 (baseline data)

#### SRB-related indicators

We measured the degree of SRB in the COVID-19 pandemic, the degree of motivation for SRB, stigma toward COVID-19 (both stigma toward others and self-stigma), anxiety in COVID-19, and depressive feelings in COVID-19 (Table 1). All the measures are the original scales developed in our previous study (Katsuki et al., 2021). The degree of SRB ranges 2 to 8, asking actual self-confinement behavior. The degree of motivation for SRB was assessed (score range 7 to 28) by asking for reasons that resulted (or did not result) in a respondent’s willingness to behave. Stigma toward SRB means stigmatized feelings and attitude toward other people (score ranges 5 to 20) and self-stigma (score ranges 9 to 36). Anxiety (score ranges 6 to 24) and depressive feelings (score ranges 4 to 16) refer to the COVID-19 related mental health alternation.

**Table 1:** Questions about self-restraint behavior (SRB), motivation for SRB (m-SRB) and COVID-19-related factors

[The following instruction and sentences were shown to the respondents.] How much has COVID-19 pandemic affected your daily life? At present as of June 2020, what are your thoughts and feelings about the following items?				
	Strongly disagree	Disagree	Agree	Strongly agree
1. I proactively avoid direct social interaction	1	2	3	4
2. I proactively refrain from going out of the house	1	2	3	4
3^†^. There is a limit to my patience, so I think it is better to act as freely as possible	1	2	3	4
4^†^. I think I can go anywhere I want because there are no punitive laws with movement restriction	1	2	3	4
5. I think we need to refrain from moving because the whole people in Japan are putting up with it	1	2	3	4
6^†^. In order to relieve stress, I think it's unavoidable to behave in a way that breaks the rule of avoiding "three Cs"	1	2	3	4
7^†^. Even if there's a chance I’m infected with COVID-19, I still want to go out and take a break	1	2	3	4
8^†^. Even if there's a chance I’m infected with COVID-19, I still want to see people for a break	1	2	3	4
9. To prevent the spread of COVID-19, I think it's necessary to refrain from moving	1	2	3	4
10. I don't want to be around people who have coughs, fevers and other cold symptoms	1	2	3	4
11. I don't want to be around people from foreign countries or domestic epidemic areas of COVID-19	1	2	3	4
12. I don't want to be anywhere near health care workers	1	2	3	4
13. If I know from the license plate number that the car came from an epidemic area, I get nervous I'm going to be exposed to COVID-19	1	2	3	4
14. If I know from the license plate number that the car is from an epidemic area, I feel "Restrain yourself and don't come here!" and get annoyed	1	2	3	4
15. If I have to go to an area where COVID-19 is not prevalent, I am worried that I will be discriminated against (*For respondents who do not live in an endemic area, please assume that you live in an endemic area)	1	2	3	4
16. If I have to go to an area where COVID-19 is not prevalent, I don't want people to know that I am from an endemic area (*For respondents who do not live in an endemic area, please assume that you live in an endemic area)	1	2	3	4
17. I'm afraid of what people around me will say if I come down with cough, fever, or other symptoms that suggest I'm infected with COVID-19	1	2	3	4
18. I want to have a PCR test as soon as I have symptoms of suspected infection with COVID-19	1	2	3	4
19. I would hesitate to see a doctor even if they have symptoms that suggest COVID-19 infection	1	2	3	4
20. If I got infected with COVID-19, I would not want people to know about it	1	2	3	4
21. If I got infected with COVID-19, I think it would cause troubles to my family and people close to me	1	2	3	4
22. If I got infected with COVID-19, I think people around would have prejudice against me	1	2	3	4
23. If I got infected with COVID-19, I think people would have prejudice against my family and others close to me	1	2	3	4
24. I’m scared of getting infected with COVID-19 from people around me	1	2	3	4
25. I'm scared of transmitting COVID-19 to others	1	2	3	4
26. I'm worried about my job status (employment, termination, leave of absence, job change, etc.)	1	2	3	4
27. I'm worried about my money (income, guaranteed absence from work, etc.)	1	2	3	4
28. I'm worried about my physical health	1	2	3	4
29. I'm worried about my mental health	1	2	3	4
30. I feel tired	1	2	3	4
31. I feel irritated and angry	1	2	3	4
32. I feel depressed	1	2	3	4
33. I feel lonely	1	2	3	4

The degree of self-restraint behavior: 1, 2

The degree of motivation for self-restraint behavior: 3, 4, 5, 6, 7, 8, 9

COVID-19-related stigma: 10, 11, 12, 13, 14

COVID-19-related self-stigma: 15, 16, 17, 18, 19, 20, 21, 22, 23

COVID-19-related anxiety: 24, 25, 26, 27, 28, 29

COVID-19-related depressive feelings: 30, 31, 32, 33

^†^ Item was reverse-scored

#### Mental health associated with hikikomori

##### **Severity of hikikomori tendency:**

the 25-item Hikikomori Questionnaire (HQ-25) (Teo et al., 2018)

The HQ-25 is a 25-item self-administered questionnaire measuring hikikomori tendencies. The questionnaire consists of three subscales related to the psychological characteristics of hikikomori. Each subscale is Lack of socialization (avoidance of social interaction), Isolation, and Lack of emotional support. It is based on a 5-point Likert scale, with respondents choosing from the following options; (0) strongly disagree (1) somewhat disagree (2) neither agree nor disagree (3) somewhat agree (4) strongly agree. Scores range from 0 to 100, with higher scores indicating more avoidance of relationships with others and higher hikikomori tendencies. Internal consistency, test–retest reliability, and convergent validity have been confirmed. The Cronbach’s alpha coefficient for the overall scale is 0.96, and the subscales are 0.94 for Lack of socialization, 0.91 for Isolation, and 0.88 for Lack of emotional support, respectively (Teo et al., 2018).

##### Severity of depressive tendency:

the Patient Health Questionnaire-9 (PHQ-9) (Muramatsu et al., 2007) and The Center for Epidemiologic Studies Depression Scale (CES-D) (Shima et al., 1985)

The PHQ-9 is a 9-item self-administered questionnaire used to screen for depression; it is a 4-point Likert scale and respondents respond from (0) not at all to (3) nearly every day for depressive symptoms over the past 2 weeks. Scores range from 0 to 27, with 5–9 points indicating mild symptoms, 10–14 moderate, 15–19 moderate to severe, and 20–27 severe. The Japanese version of the scale has been confirmed to be highly accurate, with a sensitivity of 0.84, specificity of 0.95, positive predictive value of 0.87, negative predictive value of 0.94, and kappa coefficient of 0.79 (Muramatsu et al., 2007).

The CES-D is a clinically widely used self-rating scale of depressive symptoms, consisting of 20 items. The number of days with symptoms in the past week is selected from (0) none, (1) 1–2 days, (2) 3–4 days, and (3) 5 or more days. Scores range from 0 to 60, with higher scores indicating a higher degree of depressive symptoms. The Japanese version of the scale has been confirmed to have high reliability (r = 0.84 for the retest method and rt = 0.79 for the split-half method) and have good concurrent validity (Shima et al., 1985).

##### Modern-type depression (MTD) tendency:

the 22-item Tarumi’s modern-type depression trait scale; Avoidance of social roles, Complaint and low Self-esteem (TACS-22) (Kato et al., 2019a, b) and the Interpersonal Sensitivity/Privileged Self Scale (IPS) (Muranaka et al., 2017)

Two leading scales measuring MTD tendencies were administered. The TACS-22 is a 22-item self-administered questionnaire consisting of three subscales. Each subscale is Avoidance of social roles, Complaint, and Low self-esteem; it is a 5-point Likert scale and respondents select from (0) disagree to (4) agree. Scores range from 0 to 88, with higher scores indicating higher MTD psychological characteristics. Cronbach’s alpha coefficients indicate the overall scale = 0.80, Avoidance of Social Roles = 0.74, Complaint = 0.75, Low Self-Esteem = 0.64. Convergent validity has also been confirmed (Kato et al., 2019a, b).

The IPS is a 25-item self-administered questionnaire with two subscales. The subscale consists of the two superordinate factors of interpersonal sensitivity, including evaluation apprehension, overreaction to negative feedback and avoidance, and privileged self, including sense of victimization, self-righteousness, and results dependence. 5-point Likert scale, with respondents responding from (1) not applicable to (5) applicable. Scores range from 25 to 125, with higher scores indicating stronger psychological characteristics for each. The test–retest reliability of the IPS was r = 0.82 for the evaluation apprehension score, r = 0.77 for the overreaction to negative feedback score, r = 0.69 for the sense of victimization score, and r = 0.61 for the self-righteousness score. The scale shows adequate retest reliability and has been well validated (Muranaka et al., 2017).

##### Social anxiety tendency:

the MINI-Social Phobia Inventory (MINI-SPIN) (Nagata et al., 2013)

The MINI-SPIN is a self-administered questionnaire for screening of social phobia: a 5-item Likert scale consisting of 3 items and respondents select from (0) not at all to (4) extremely for symptoms in the past week. Scores range from 0 to 12, with a cutoff value of 6 (Connor et al., 2001). The Japanese version of the scale (SPIN-J) reported a Cronbach’s alpha coefficients of 0.96, which is highly reliable and well validated (Nagata et al., 2013).

##### Internet addiction tendency:

Young’s Internet Addiction Test (IAT) (Mak et al., 2014).

The IAT is a 20-item, self-administered questionnaire regarding frequency and excessive use of the Internet, using a 6-point Likert scale, with respondents responding on a scale of (0) not applicable to (5) always regarding their Internet use experience. Scores range from 0 to 120, with higher scores indicating a higher degree of problematic use of the Internet (Young, 1998). The Japanese version of the scale has been validated and Cronbach's alpha coefficients reported as 0.85 (Lai et al., 2015; Mak et al., 2014).

##### Motivation:

Achievement Motivation Scale (Horino & Mori, 1991)

The Achievement Motivation Scale is a 24-item self-administered questionnaire that measures two achievement motives. Competitive Achievement Motivation is the motivation to outperform and beat others in order to be appreciated by society and Self-fulfilling Achievement Motivation is the motivation to reach his/her own standards of achievement without regard to the evaluations of others or society. It is a 7-point Likert scale, and respondents answer on a scale of (1) not at all to (7) very much. The score range is 24 to 168, with higher scores indicating a higher achievement motivation. Reliability coefficients using the Spearman-Brown split-half formula were rt = 0.91 for the competitive achievement motivation and rt = 0.87 for the self-fulfilling achievement motivation, confirming the reliability and validity of the scale (Horino & Mori, 1991).

##### Resilience:

Tachikawa resilience scale (TRS) (Nishi et al., 2013)

The TRS is a self-administered questionnaire modified to the Japanese culture to measure resilience. This scale consists of 10 items on a 7-point Likert scale, with respondents responding from (1) strongly disagree to (7) strongly agree. Scores range from 10 to 70, with higher scores indicating greater resilience. Regarding reliability, the Cronbach's alpha coefficient is 0.82. Validity has also been confirmed (Nishi et al., 2013).

#### (2) Questionnaires implemented in December 2020

#### Hikikomori status

We asked the number of days spent on going out for more than one hour per week. Since those who are able to go out for limited periods of less than one hour are also included in hikikomori, we asked how often they go out for more than one hour (Kato et al., 2020a; Saito, 2010; Teo et al., 2015a).

##### Severity of hikikomori tendency:

HQ-25

#### Mental health status

##### Severity of depressive tendency:

PHQ-9 and CES-D

##### Modern-type depression (MTD) tendency:

TACS-22 and IPS

##### Internet addiction tendency:

IAT

#### Statistics

Due to normality deviations from the normal distribution of the obtained data, robust methods were employed in the subsequent analysis. First, for SRB-related indicators (Katsuki et al., 2021), we used data on 800 participants’ data obtained in December 2020 to perform a confirmatory factor analysis (CFA) with diagonally weighted least squares (DWLS) method. DWLS is recommended when non-normal or categorical data are included in the observed variables (Toyoda, 2014). The results showed a factor structure consistent with previous studies (Katsuki et al., 2021) as shown in Table 2 (χ^2^ (480) = 3532.99, *p* < 0.01, CFI = 0.87, TLI = 0.86, RMSEA = 0.09, SRMR = 0.11). The Internal consistencies (Cronbach’s alpha) for the factors ranged from 0.72 (COVID-19-related stigma) to 0.86 (COVID-19-related depressive feelings). Therefore, subsequent analysis was based on the factor structure.

**Table 2:** SRB-related indicators: Standardized Factor Loadings for the CFA Model and Cronbach’s coefficient alpha

	F1	F2	F3	F4	F5	F6	
F1. The degree of self-restraint behavior (α = 0.74)							
1. I proactively avoid direct social interaction	0.81						
2. I proactively refrain from going out of the house	0.72						
F2. The degree of motivation for self-restraint behavior (α = 0.82)							
3^†^. There is a limit to my patience, so I think it is better to act as freely as possible		0.63					
4^†^. I think I can go anywhere I want because there are no punitive laws with movement restriction		0.72					
5. I think we need to refrain from moving because the whole people in Japan are putting up with it		-0.60					
6^†^. In order to relieve stress, I think it's unavoidable to behave in a way that breaks the rule of avoiding "three Cs"		0.54					
7^†^. Even if there's a chance I’m infected with COVID-19, I still want to go out and take a break		0.54					
8^†^. Even if there's a chance I’m infected with COVID-19, I still want to see people for a break		0.53					
9. To prevent the spread of COVID-19, I think it's necessary to refrain from moving		-0.68					
F3. COVID-19-related stigma (α = 0.72)							
10. I don't want to be around people who have coughs, fevers and other cold symptoms			0.62				
11. I don't want to be around people from foreign countries or domestic epidemic areas of COVID-19			0.68				
12. I don't want to be anywhere near health care workers			0.40				
13. If I know from the license plate number that the car came from an epidemic area, I get nervous I'm going to be exposed to COVID-19			0.44				
14. If I know from the license plate number that the car is from an epidemic area, I feel "Restrain yourself and don't come here!" and get annoyed			0.43				
F4. COVID-19-related self-stigma (α = 0.85)							
15. If I have to go to an area where COVID-19 is not prevalent, I am worried that I will be discriminated against (*For respondents who do not live in an endemic area, please assume that you live in an endemic area)				0.71			
16. If I have to go to an area where COVID-19 is not prevalent, I don't want people to know that I am from an endemic area (*For respondents who do not live in an endemic area, please assume that you live in an endemic area)				0.75			
17. I'm afraid of what people around me will say if I come down with cough, fever, or other symptoms that suggest I'm infected with COVID-19				0.80			
18. I want to have a PCR test as soon as I have symptoms of suspected infection with COVID-19				0.40			
19. I would hesitate to see a doctor even if they have symptoms that suggest COVID-19 infection				0.31			
20. If I got infected with COVID-19, I would not want people to know about it				0.68			
21. If I got infected with COVID-19, I think it would cause troubles to my family and people close to me				0.57			
22. If I got infected with COVID-19, I think people around would have prejudice against me				0.74			
23. If I got infected with COVID-19, I think people would have prejudice against my family and others close to me				0.68			
F5. COVID-19-related anxiety (α = 0.85)							
24. I’m scared of getting infected with COVID-19 from people around me					0.66		
25. I'm scared of transmitting COVID-19 to others					0.59		
26. I'm worried about my job status (employment, termination, leave of absence, job change, etc.)					0.68		
27. I'm worried about my money (income, guaranteed absence from work, etc.)					0.67		
28. I'm worried about my physical health					0.80		
29. I'm worried about my mental health					0.79		
F6. COVID-19-related depressive feelings (α = 0.86)							
30. I feel tired						0.85	
31. I feel irritated and angry						0.80	
32. I feel depressed						0.83	
33. I feel lonely						0.61	
	Factor	Factor correlations					
F1. The degree of self-restraint behavior	F1	―					
F2. The degree of motivation for self-restraint behavior	F2	-0.33	―				
F3. COVID-19-related stigma	F3	0.73	-0.25	―			
F4. COVID-19-related self-stigma	F4	0.50	-0.21	0.71	―		
F5. COVID-19-related anxiety	F5	0.55	-0.05	0.58	0.60	―	
F6. COVID-19-related depressive feelings	F6	0.49	0.14	0.48	0.45	0.78	―

^†^ Item was reverse-scored

In addition to hikikomori tendency evaluating with HQ-25, we assessed the physical hikikomori using data of actual outings in December 2020 for participants who went out more than four days per week in June 2020. Of 800 participants, 402 were going out more than four days per week in June 2020. Among them, 63 participants were going out less than one day per week (i.e., Lower going out group); 339 participants were going out more than two days per week (i.e., Higher going out groups) in December 2020. Mann–Whitney *U* test was implemented to compare each subscale of HQ-25 and mental health status between Lower and Higher going out groups in December 2020.

A cross-lagged effects model was performed to examine whether SRB can predict hikikomori and the association between SRB, hikikomori, and mental health using self-administered scale data obtained at two time points, June 2020 and December 2020. Since the observed variables are non-normal, we incorporated a MLM as estimator, which is a maximum likelihood estimation method with robust standard errors and a Satorra-Bentler scaled test statistic (Rosseel, 2014). The model included three subscales of HQ-25, three subscales of TACS-22, two subscales of IPS, PHQ-9, CES-D, and IAT; MINI-SPIN, Achievement Motivation Scale, and TRS were not included in the model, not administered in December 2020.

Group comparison was performed with IBM SPSS 24 Advanced Statistics for Mac OS. The CFA for SRB-related indicators and cross-lagged effects model analysis were conducted by R version 4.1.2 (R Core Team, 2021) using the lavaan package (Rosseel, 2012).

## Results

Comparing the 247 participants who did not participate in the study (dropout group) with the 800 participants with no missing values, the dropout group (M = 43.8, SD = 8.4) was younger than the participant group (M = 45.5, SD = 8.2) (Mann–Whitney *U* test, *z* = -3.25, *p* < 0.01). Gender was not significantly different between the dropout group (male, 172; female, 206) and the participant group (male, 401; female, 399) (χ^2^ (1) = 2.20, N.S.). Also, in June 2019 (one year before the baseline data obtention), all of both groups were employed, but in June 2020, more unemployed individuals were observed in the dropout group (working, 348; not working, 21; housewife/housekeeper, 9) than the participant group (working, 772; not working, 23; housewife/housekeeper, 5). Hence, the dropout group possibly included more people with hikikomori (χ^2^ (1) = 12.13, *p* < 0.01). There were no significant differences in the extent of the COVID-19 epidemic by residence or work place (χ^2^ (1) = 3.14, N.S.; χ^2^ (1) = 0.58, N.S.).

Of the 402 participants who went out more than four days per week in June 2020, 339 were stayed in the Higher going out group (going out more than two days per week) and 63 were shifted to the Lower going-out group (going out less than one day per week) as of December 2020. The demographic data and online survey scores for both groups in December 2020 are shown in Table 3. In both groups, most of the participants were employed. There were no significant differences in measures evaluating mental health status.

**Table 3:** Demographic characteristics and self-rated questionnaires of participants in Dec. 2020 (Data limited to participants who went out more than four days per week in Jun. 2020; Total *N* = 402)

	Lower going out (Going out less than one day per week)		Higher going out (Going out more than two days per week)		*p* value (Mann–Whitney *U* test)
Total	63		339		-
Male/Female	26/37		155/184		-
Work status					
Employed/Self-employed	63		336		-
Without occupation	0		2		-
House manager	0		1		-
	Mean	*SD*	Mean	*SD*	
Age	46.37	*7.96*	45.6	*8.22*	0.56
Hikikomori tendency					
HQ-25 sub-scale					
Lack of Socialization	23.73	*10.20*	24.73	*9.64*	0.46
Isolation	13.65	*7.87*	12.72	*6.41*	0.26
Emotional Support	9.68	*5.90*	9.83	*5.80*	0.74
Depressive symptoms					
PHQ-9	4.71	*5.44*	5.15	*5.34*	0.37
CES-D	13.24	*8.78*	13.84	*9.72*	0.89
MTD tendency					
TACS-22 sub-scale					
Avoidance	22.02	*6.12*	23.32	*5.91*	0.18
Low Self-esteem	10.33	*4.62*	10.80	*4.46*	0.49
Complaint	7.10	*4.45*	7.67	*5.22*	0.52
IPS sub-scale					
Interpersonal Sensitivity	32.43	*8.58*	33.38	*9.03*	0.39
Privileged Self	29.63	*8.06*	29.11	*7.78*	0.33
Internet Addiction					
IAT	27.24	*8.75*	29.78	*10.41*	0.09†
SRB-related indicators					
Degree of SRB	4.98	*1.62*	4.84	*1.53*	0.45
Motivation for SRB	21.92	*3.80*	22.08	*4.00*	0.69
COVID-19-related stigma	12.19	*2.84*	11.87	*2.73*	0.18
COVID-19-related self-stigma	23.19	*5.26*	24.18	*4.67*	0.11
COVID-19-related anxiety	15.60	*4.53*	15.49	*3.96*	0.70
COVID-19-related depressive feelings	9.03	*3.24*	9.08	*2.95*	0.96

† *p* < 0.10

**Fig. 1:**
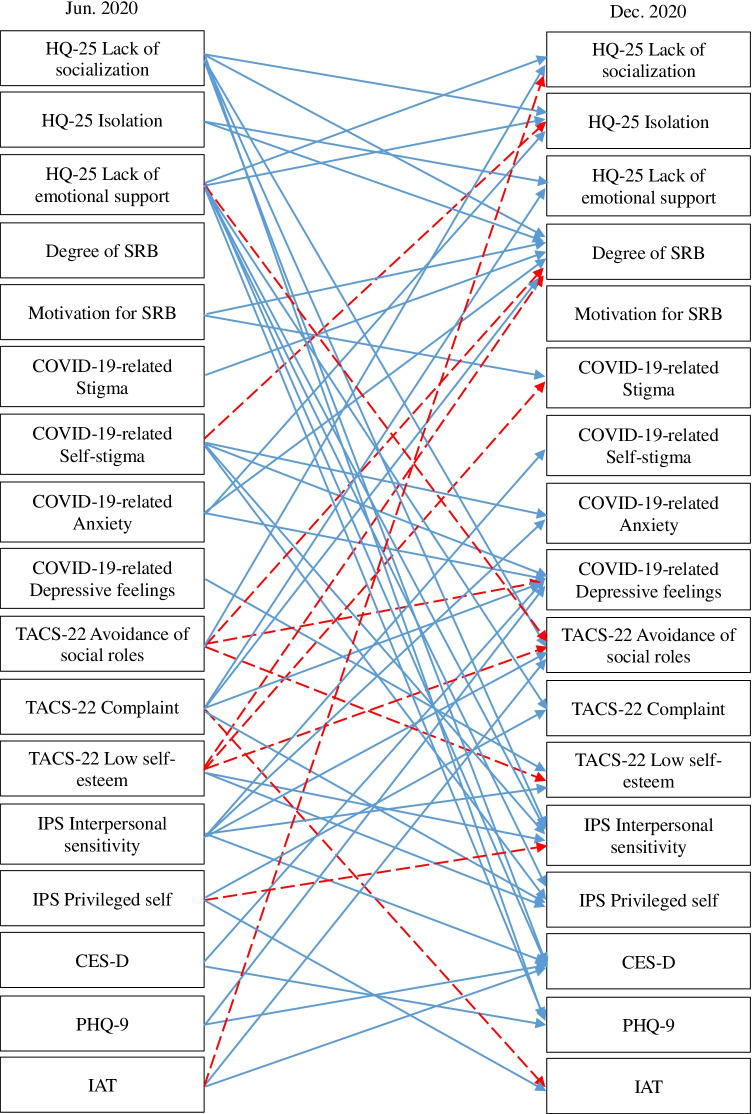
Cross-lagged effects models (All variables). Note: Same-variable and nonsignificant paths, error factors, and covariance among variables were omitted

**Fig. 2:**
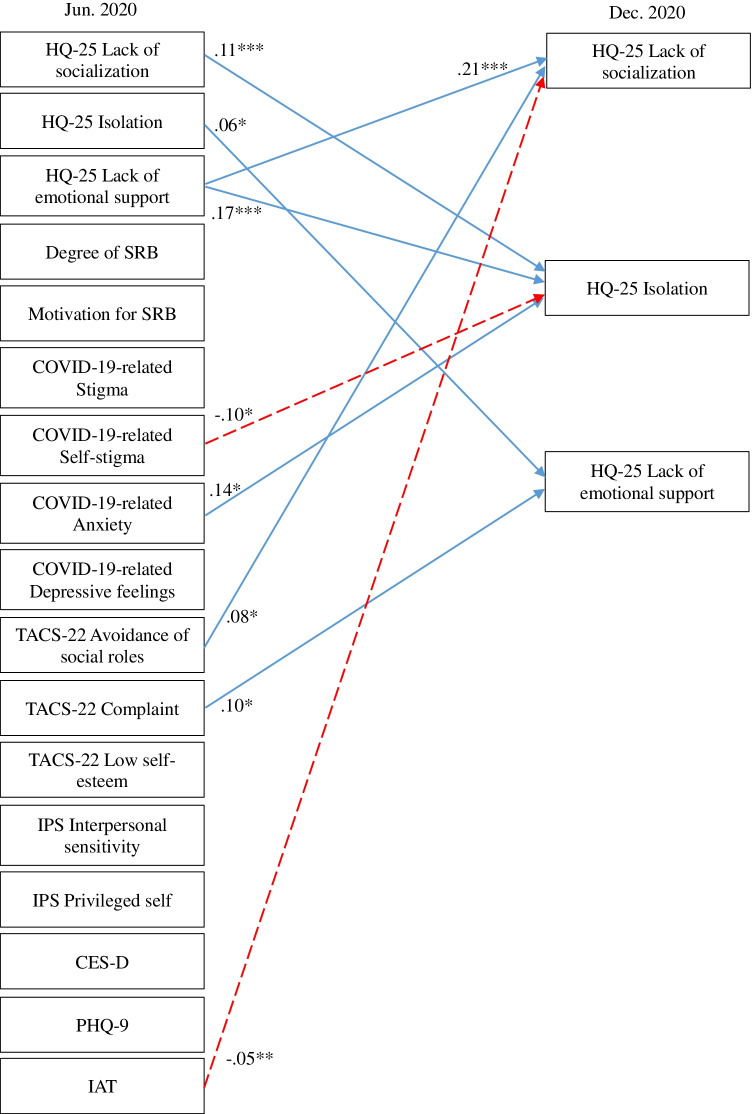
Cross-lagged effects models (Hikikomori-related variables). Note: Hikikomori-related variables were extracted from Fig. 1. For simplicity, same-variable and non-significant paths, error factors, and covariance among variables were omitted. * *p* < 0.05, ** *p* < 0.01, *** *p* < 0.001

**Fig. 3:**
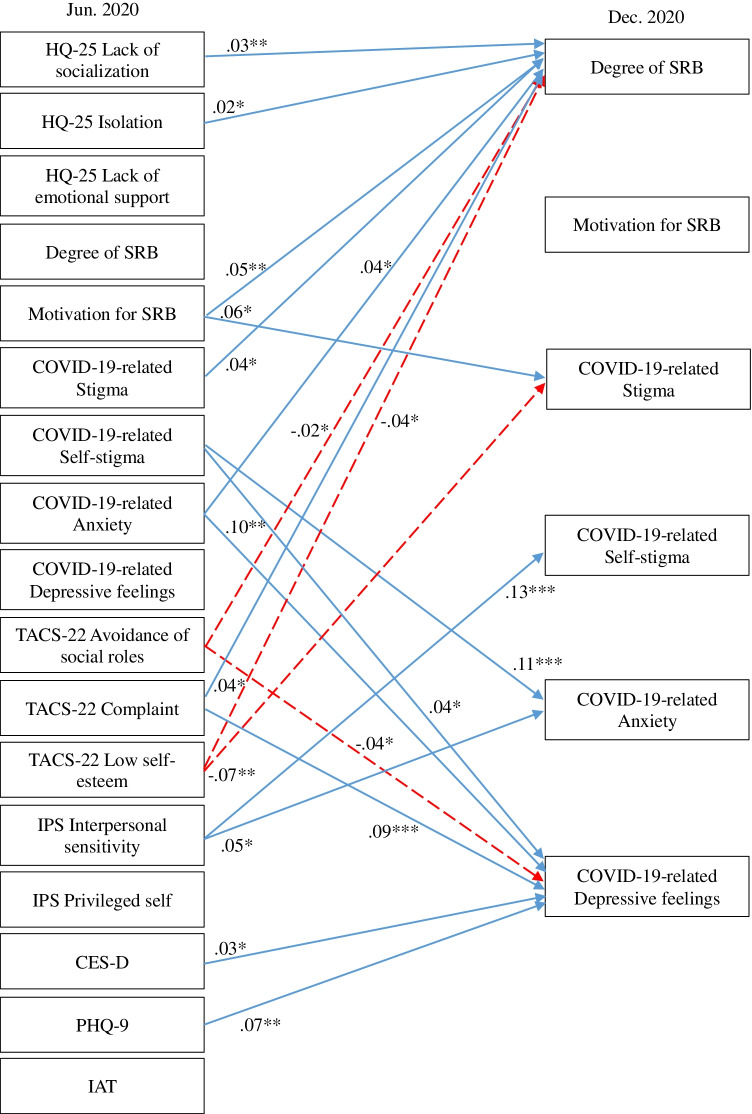
Cross-lagged effects models (SRB-related variables). Note: SRB-related variables were extracted from Fig. 1. For simplicity, same-variable and non-significant paths, error factors, and covariance among variables were omitted. * *p* < 0.05, ** *p* < 0.01, *** *p* < 0.001

**Fig. 4:**
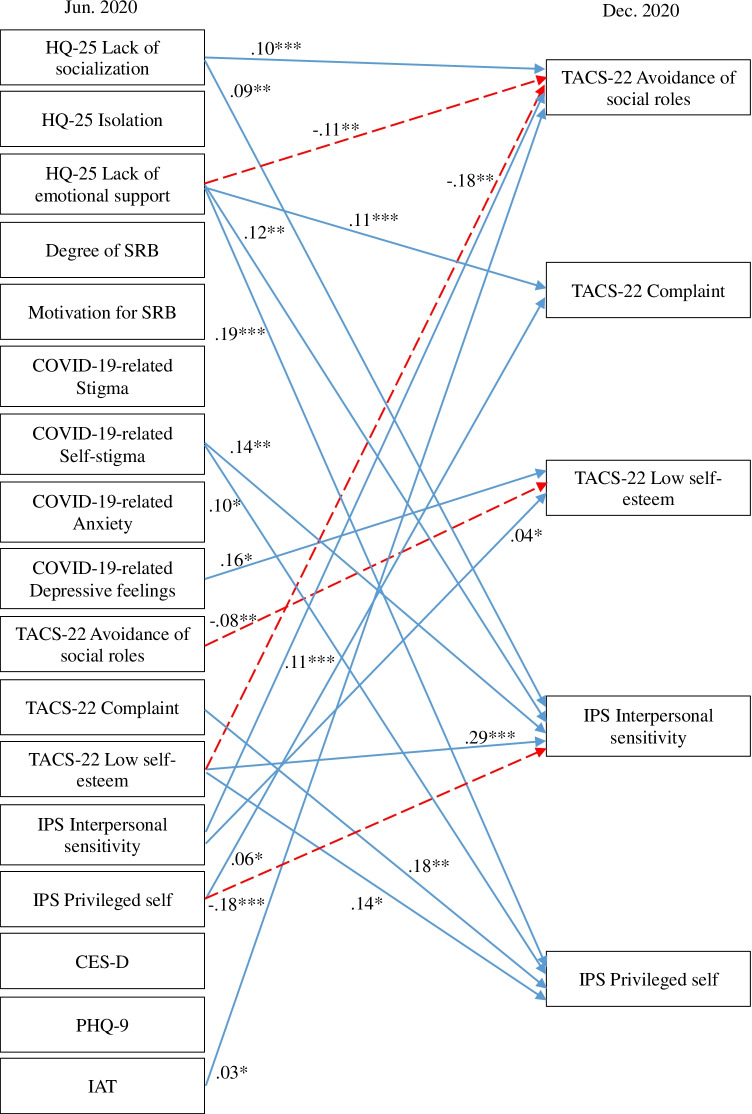
Cross-lagged effects models (MTD-related variables). Note: MTD-related variables were extracted from Fig. 1. For simplicity, same-variable and non-significant paths, error factors, and covariance among variables were omitted. * *p* < 0.05, ** *p* < 0.01, *** *p* < 0.001

**Fig. 5:**
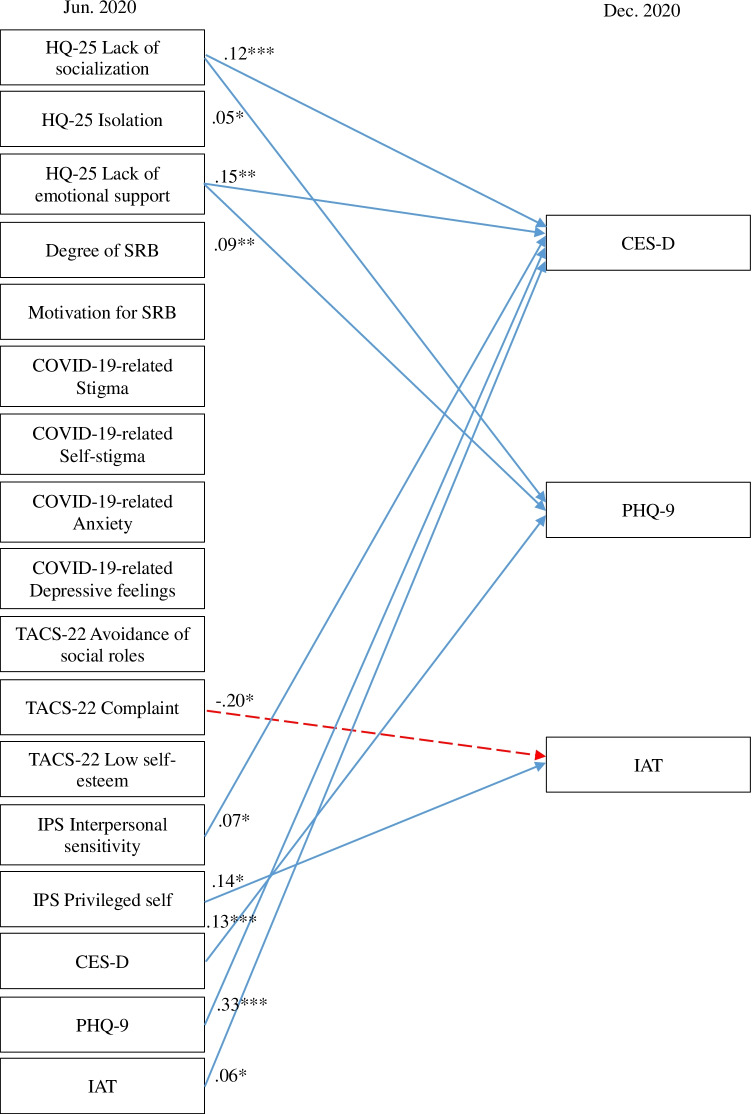
Cross-lagged effects models (Other mental health-related variables). Note: Other mental-health-related variables were extracted from Fig. 1. For simplicity, same-variable and non-significant paths, error factors, and covariance among variables were omitted. * *p* < 0.05, ** *p* < 0.01, *** *p* < 0.001

A cross-lagged effects model showing the association between SRB, hikikomori, and mental health conditions is shown in Fig. 1 (all variables) and Figs. 2, 3, 4, and 5 (excerpts for hikikomori-related, SRB-related, MTD-related, and other mental health-related variables, respectively). The model was a saturated model, with goodness-of-fit indices of χ^2^ (0) = 0, CFI = 1.00, TLI = 1.00, AIC = 148,516.47, RMSEA = 0.00, SRMR = 0.00.

For the three subscales of HQ-25 (Fig. 2), lack of emotional support had a positive cross-lagged effect on lack of socialization, while IAT showed a negative cross-lagged effect. In addition, lack of socialization and TACS-22 avoidance of social roles had a positive cross-lagged effect with each other. On isolation, lack of socialization and the SRB-related indicators of COVID-19-related anxiety showed a positive cross-lagged effect, and COVID-19-related self-stigma showed negative a cross-lagged effect. Isolation and lack of emotional support also showed a positive cross-lagged effect with each other. TACS-22 complaint had a positive cross-lagged effect on lack of emotional support.

Regarding SRB-related indicators (Fig. 3), degree of SRB was influenced with positive cross-lagged effects of many variables, including not only motivation for SRB, but also HQ-25 lack of socialization and isolation, COVID-19-related stigma and anxiety, and TACS-22 complaint. SRB was also affected by negative cross-lagged effects of TACS-22 avoidance of social roles and low self-esteem. On the other hand, SRB itself had no significant cross-lagged effect on any of the other variables. COVID-19-related anxiety was influenced with a positive cross-lagged effect from COVID-19-related self-stigma and IPS interpersonal sensitivity; and COVID-19-related self-stigma was affected by a positive cross-lagged effect from IPS interpersonal sensitivity. COVID-19-related depressive feelings were affected by positive cross-lagged effects of COVID-19-related anxiety and self-stigma, CES-D, PHQ-9, and TACS-22 complaint, and were affected by a negative cross-lagged effect from TACS-22 avoidance of social roles.

As for MTD-related measures (Fig. 4), IPS privileged self and IAT showed positive cross-lagged effects on TACS-22 avoidance of social roles, while HQ-25 lack of emotional support showed negative cross-lagged effects. TACS-22 complaint showed a positive cross-lagged effect with HQ-25 lack of emotional support and IPS privileged self, and a negative cross-lagged effect with low self-esteem and each other, as noted earlier. Low self-esteem had a positive cross-lagged effect on IPS interpersonal sensitivity and privileged self. IPS interpersonal sensitivity was affected by positive cross-lagged effects by HQ-25 lack of socialization and lack of emotional support, suggesting a complex relationship between hikikomori and MTD.

For other mental health measures (Fig. 5), the CES-D received positive cross-delay effects from IPS interpersonal sensitivity and IAT as well as HQ-25 lack of socialization and lack of emotional support. The PHQ-9 similarly received a positive cross-lagged effect from lack of socialization and lack of emotional support on the HQ-25 subscale.

## Discussions

In the present study, we conducted a prospective online survey with a six-month interval to examine the effects of self-restraint behavior (SRB) on hikikomori in the COVID-19 pandemic. In addition to SRB, we also examined mental health problems such as depression, MTD, and Internet addiction tendencies. A cross-lagged effects model was performed to evaluate the interrelationships among each variable obtained at six-month intervals (Figs. 1, 2, 3, 4, and 5). As a result, significant paths were revealed for each variable. Based on these results, four main perspectives are discussed.

1. **Variables affecting HQ-25**
  First, association were found for the three subscales of HQ-25. Lack of emotional support was found to have a positive cross-lagged effect on lack of socialization at second time point (6 months); lack of socialization was found to have a positive cross-lagged effect on isolation at 6 months. Lack of emotional support was also found to interact with isolation, suggesting that office workers with less emotional support and less socialization at June 2020 were more isolated six months later, in December 2020. Previous studies pointed out that introversion and interpersonal problems are risk factors for hikikomori (Chong & Chan, 2012; Yong & Nomura, 2019; Kato et al., 2019b). In addition, problematic coping such as lack of help-seeking were noted in hikikomori cases (Kondo et al., 2013; Nonaka & Sakai, 2021). Association among isolation and lack of socialization and emotional support would support the previous finding that introducing functional coping, such as appropriate emotional support, can help prevent or improve hikikomori (Akbari et al., 2021; Nonaka & Sakai, 2021).
  Interestingly, we also found that the IAT had a negative cross-lagged effects on lack of socialization, suggesting that Internet use may have a protective effect on socialization. Previous research has reported that the higher the propensity for Internet addiction, the higher the tendency for hikikomori (Tateno et al., 2019). The differences between the previous and the present study were presumably influenced by differences in Internet usage. The previous study examined the relationship between online game and SNS usage and hikikomori tendencies among college students (Tateno et al., 2019). On the other hand, the present study targeted office workers in their 30 s or older, and to some extent, respondents are assumed to participate in social activities using the Internet such as remote work. Although IAT measures pathological Internet use, Internet use in remote work is a necessary usage and was not thought to be pathological usage in this study. The present result that keeping social connections through remote work with Internet did not worsen social deficits, even if going out is restricted, may suggest that the Internet use may not necessarily be harmful (Sakamoto & Saku, 2021). However, the present study did not examine the Internet use including remote work in detail. In addition, cross-lagged effects model suggests that IAT may increase the depressive tendency in CES-D and avoidant tendency in TACS-22, therefore the impact of the Internet on overall mental health should be carefully examined. COVID-19 pandemic is considered to continue the restraint of going out, and establishing a lifestyle that utilizes the Internet in a healthy way would be one solution. According to a study examining the relationship between hikikomori and the Internet in the COVID-19 pandemic, stay-home increased the number of days of hikikomori and the time spent on the Internet. However, this study did not conduct a detailed data analysis due to the small sample size (Higuchi et al., 2020). Therefore, further research on the way of using the Internet under the COVID-19 pandemic will be required. COVID-19 pandemic has firmly positioned the Internet as a more essential life tool; it may be time to revise the conventional view that excessive Internet use immediately leads to pathological addiction. The present result would support the idea.
  SRB-related psychological indicators suggested to influence HQ-25 isolation; COVID-19-related anxiety increased isolation, while COVID-19-related self-stigma reduced isolation. Although self-stigma has been suggested to increase COVID-19-related anxiety, those with basically higher anxiety to COVID-19 were higher isolation, and conversely, those with higher self-stigma were lower isolation. More generalized anxiety such as anxiety for COVID-19 may promote retreat from interpersonal relationships (Kato et al., 2020b; Rooksby et al., 2020; Wong, 2020). On the other hand, self-stigma measured in this study assumed to be related to awareness to others, such as concern about infecting others or being a nuisance. Therefore, increased self-stigma may lead people to be more conscious of others and reduce their tendency to isolate themselves from others. Alternatively, people who are more conscious of others may have higher self-stigma. These hypotheses need to be further explored in the future study.
2. **Variables affecting SRB**
  For SRB, motivation for SRB led to actual SRB six months later, consistent with previous research showing that motivation for SRB influences actual changes in outgoing behavior (Katsuki et al., 2021). Furthermore, the present results suggest that SRB may have been caused not only by awareness of the social change of the novel COVID-19 pandemic, but also by lack of socialization and complaints, as well as an original tendency toward isolation, which led to retreat from interpersonal relationships and consequently resulting in SRB.
  In this study, MTD tendencies were measured by the TACS-22 and IPS. While the TACS-22 infers that the complaint subscale increases SRB, two subscales, increased avoidance of social roles and lowered self-esteem, suggesting that SRB is reduced by the MTD tendency. The characteristic of MTD is avoidance of roles and responsibilities demanded by society (Kashihara et al., 2019; Kato & Kanba, 2017; Kato et al., 2016a, b). SRB in the COVID-19 pandemic was requested by the government in Japan, and such the tendency not to follow social demands may have hindered SRB. On the other hand, for MTD tendencies measured using the IPS, the IPS did not predict SRB. However, interpersonal sensitivity, a subscale of the IPS, increased COVID-19-related anxiety, a factor in SRB. Although not fully elucidated in the present study, interpersonal sensitivity may be a factor in defining affinity for social roles and warrants further investigation.
3. **Relationship between MTD and hikikomori**
  The cross-lagged effects model suggested a complex relationship between hikikomori and MTD; tendency to avoid social roles in TACS-22 and lack of socialization in HQ-25 interacted with each other. Similarly, complaint in TACS-22 and lack of emotional support in HQ-25 interacted. Lack of emotional support was suggested to increase both interpersonal sensitivity and privileged self in IPS. These results are the first empirical evidence supporting the hypothesis that MTD is a gateway condition to hikikomori (Kato & Kanba, 2017) and suggest that hikikomori may also be a gateway to MTD. Although the causal relationship is not clear, the lack of adequate support in neighbor relationships increases the tendency in complaint and privileged self, leading to reduced social skills and retreat from social roles, which further leads to poor interpersonal relationships. It is speculated that this vicious cycle may lead to hikikomori tendencies and MTD. Interestingly, on the other hand, lack of emotional support was suggested to reduce the tendency to avoid social roles six months later. Lack of emotional support seems to interact with isolation and increase interpersonal sensitivity. These results suggest that lack of emotional support may lead to relationship-seeking, which may include a protecting disposition such as amae (Kato et al., 2012). Further studies are needed to clarify the role of emotional support among hikikomori and MTD.
4. **Relationship between hikikomori, SRB and mental health**
  The comorbidity of hikikomori and depression has been repeatedly reported from epidemiological studies (Kato et al., 2012; Kondo et al., 2013; Koyama et al., 2010; Teo et al., 2015b, 2020). In common with the CES-D and PHQ-9 scores in this study, lack of socialization and emotional support on the HQ-25 increased these depressive scores after six months. These results suggest that hikikomori tendencies may increase depressive symptoms. An important finding of this study is that the SRB itself did not show significant effects on other mental health including depressive symptoms. In other words, SRB itself is unlikely to be a factor in worsening hikikomori risk nor mental health. On the other hand, as noted above, the plausible relationship between hikikomori and depressive tendency suggests that early intervention to hikikomori may be required to prevent deterioration of mental health in COVID-19 pandemic. In particular, given the present findings that interpersonal problems such as lack of emotional support and interpersonal sensitivity are interrelated with hikikomori, the promotion of social connection and emotional support will be important to prevent the transition to pathological hikikomori (Li et al., 2020; Tull et al., 2020; Miki et al., 2021). In addition, considering the previous study that appropriate coping, such as help-seeking, can help prevent or improve hikikomori (Akbari et al., 2021; Nonaka & Sakai, 2021), a proactive approach by professionals would be important. Given that many of the participants in this study are in a working status, such as company employees, workplace countermeasures during a COVID-19 pandemic may promote and maintain employees’ mental health and work performance (Sasaki et al., 2020). As a specific intervention targeting workplace, several interventions including resilience-building programs are proposed (Giorgi et al., 2020). Providing such accessible support may be useful in supporting hikikomori. Providing such accessible support may be useful in supporting hikikomori. However, due to the large-scale stay-home caused by COVID-19, face-to-face opportunities to assess psychosocial needs and provide support have been greatly reduced (Gaebel & Stricker, 2020; Pfefferbaum & North, 2020). To overcome such a situation, an appropriate combination of traditional face-to-face support as well as remote support using online tools could prevent the transition to pathological hikikomori in the COVID-19 pandemic (Colle et al., 2020; Gaebel & Stricker, 2020; Li & Leung, 2020; Li et al., 2020; Pignon et al., 2020). Furthermore, studies have indicated that deteriorating family relationships may intensify the mental health problems caused by COVID-19 (Stavridou et al., 2020). Thus, family support is expected to prevent mental health problems and transition to hikikomori (Kubo et al., 2020, 2021).
  One limitation of this study is that social anxiety, motivation, and resilience, as measured by the MINI-SPIN, Achievement Motivation Scale, and TRS, could not be implemented at two time points and included in the cross-lagged effects model. All of these measures may be factors that promote or prevent hikikomori, and refining the model to include these variables will be necessary in the future. In addition, although the present study was an observational study and we did not pre-register for the study protocol, publication bias and the possibility of post hoc analysis may arise, and pre-registration may be required to improve the quality of the study.
  Despite these limitations, this study elucidated some risk factors affecting hikikomori during the COVID-19 pandemic and succeeded in demonstrating empirically that MTD and hikikomori may influence each other for the first time. In addition, the results of office workers indicate that interpersonal problems such as lack of emotional support and interpersonal sensitivity are interrelated with hikikomori, and that active promotion of social connections and emotional support is required to prevent deterioration of mental health and transition to pathological hikikomori. Since hikikomori is a common phenomenon not only in Japan but also worldwide (Kato et al., 2012), conducting similar surveys as a countermeasure of hikikomori and to identify factors of hikikomori in different cultures in other countries would be important. Just recently, a cross-sectional study reported that home quarantine due to the COVID-19 pandemic was associated with depressive symptoms in China (Tang et al., 2022). Including such findings, further studies are required worldwide.

## Data Availability

The datasets generated during and/or analyzed during the current study are available from the corresponding author on reasonable request.
